# Aging-Feature-Extraction Method Based on the Short-Axis Diameter Ratio of Serviced Rubber Strips

**DOI:** 10.3390/polym18050647

**Published:** 2026-03-06

**Authors:** Yujia Chen, Bo Xu, Yun Tan, Jia He, Youchun Pi, Hu Li, Chunyu Meng, Yiyi Liang, Mengyue Bai, Yuansi Wei

**Affiliations:** 1 China Yangtze Power Co., Ltd., Yichang 443000, China; 2 Hubei Technology Innovation Center for Smart Hydropower, Wuhan 430000, China; 3 Guangzhou Mechanical Engineering Research Institute Co., Ltd., Guangzhou 510700, China

**Keywords:** rubber aging, feature analysis, short-axis diameter ratio, elastic modulus comparison, serviced rubber strips

## Abstract

In this paper, aiming at the aging problem of rubber sealing strips in key parts of hydropower units under long-term load, this study proposes a quantitative aging-feature-extraction technique centered on the ratio of the short-axis length to the original diameter (*b*/*D*) of serviced rubber strips. Through a systematic approach combining theoretical analysis, numerical simulation, and measured data calculations, the research first derives from energy principles that the elastic modulus (*E*) and yield stress (*σ_s_*) are key physical parameters characterizing rubber aging, reflecting the material’s energy storage capacity and irreversible deformation threshold, respectively. Based on this, a radial compression simulation model of rubber strips is established, focusing on the cross-sectional deformation laws under 25% and 30% compression ratios in serviced conditions. It is found that the short-axis diameter ratio *b*/*D* exhibits a significant linear relationship with the dimensionless yield stress (*σ_s_*/*E*), and a quadratic relationship with the dimensionless unit-length reaction force (*F*/*ED*). Using measured data, fluororubber (FKM) and nitrile rubber (NBR) specimens after 17 years of service are selected for radial compression experiments to extract the elastic modulus. The calculated results are compared with elasticity modulus estimates based on hardness empirical formulas (Gent’s and Qi’s formulas), showing consistency, particularly with Qi’s formula for NBR. This method enables rapid and accurate assessment of rubber aging, demonstrating the effectiveness and practicality of using *b*/*D* as a feature parameter. The study provides a quantitative and convenient tool for condition monitoring and life prediction of industrial equipment seals, especially suitable for the operation and maintenance of rubber components in complex environments such as hydropower units.

## 1. Introduction

Hydropower units are the core component of hydropower station power generation systems. Rubber seals, as an important part of hydropower units, directly impact the stability and reliability of hydropower station power generation systems [1,2,3]. Rubber seals in hydroelectric power units are subject to multiple failure mechanisms during operation. First, long-term hydraulic loads cause material fatigue and aging. This consequently results in reduced elasticity and permanent deformation. Second, mechanical vibrations induce micro-motion wear and loosening of connection components. These vibrations compromise the integrity of the sealing interface. Third, environmental corrosion accelerates surface degradation of the material. These damage mechanisms interact synergistically, leading to a continuous decline in sealing performance. This not only increases the risk of leakage but may also result in reduced turbine efficiency or even unplanned shutdowns, ultimately severely impacting the reliability and economic efficiency of hydropower plant operations [4,5,6,7,8].

To uncover the mechanisms behind the failure of rubber seals, researchers both domestically and internationally have conducted systematic studies. These studies focus on two key areas. (1) Material properties: Accelerated aging tests and molecular dynamics simulations have revealed the microscopic mechanisms underlying rubber oxidation degradation and stress relaxation. (2) Mechanical behavior: Fatigue tests and finite element analysis have been combined to elucidate the patterns of crack initiation and propagation under alternating loads. These studies have not only established a physical model for the failure of rubber seals but also provided a theoretical foundation for life prediction and performance optimization [9,10,11,12,13,14]. W. Lou et al. studied the long-term thermal-oxidative aging behavior of hydrogenated nitrile rubber seals [15]. Their study examined both uncompressed and compressed samples. The analysis was based on multiple factors. These include weight loss, chemical structure, and crosslink density. Additionally, compression deformation, fracture morphology, and mechanical properties were considered. Dong L et al. addressed the issue of seal failure in rubber tubes at high temperatures [16]. They studied the parameters of the rubber tube constitutive model through rubber thermal aging experiments and established a finite element calculation model of the packer in the seated state to investigate the effects of temperature loads on the rubber seal performance and strength behavior of the packer. Y Li et al. coupled changes in mechanical behavior with changes in the tribological behavior of NBR in oil and thermal environments to investigate the effects of oil and thermal aging on the properties of NBR seals [17]. Kömmling proposed using relaxation and recovery tests to investigate the mechanism behind the slight increase in hardness of EPDM specimens during aging, and suggested using Shore hardness and the International Rubber Hardness Scale to characterize the hardness of O-ring surfaces and cross-sections, respectively [18].

However, existing research still faces several pressing issues. On the one hand, most studies focus on single factors affecting rubber aging. However, in actual operating environments, multiple factors interact. These factors synergistically accelerate the aging process of rubber seals. Research on aging mechanisms under complex coupled factors remains insufficient. On the other hand, existing assessment models for aging and lifespan prediction have limitations. They often fail to adequately consider the nonlinear mechanical behavior of rubber under long-term loading. They also overlook the evolution of microstructure. Consequently, discrepancies exist between predicted results and actual conditions. Additionally, quantitative assessment studies on the impact of performance degradation of aged rubber seals on the overall operational reliability of hydroelectric power units are also relatively scarce.

Based on this, the present study aims to thoroughly investigate the long-term aging behavior of rubber sealing strips. These strips are located at critical positions in hydropower units. They operate under service loading conditions. The study focuses on resolving two issues. First, the aging mechanism under multi-factor coupling remains unclear. Second, existing assessment models show insufficient accuracy. By integrating theoretical analysis, numerical simulation, and measured data, the synergistic effects of hydraulic, mechanical, and environmental factors are comprehensively considered. The evolution of rubber microstructure during aging is explored—quantified by the permanent deformation parameter *b*/*D* (short-axis diameter ratio)—and a feature-extraction model centered on elastic modulus and yield stress is established. A rapid aging-characterization method based on the short-axis diameter ratio of in-service rubber strips is proposed and validated through radial compression tests and hardness comparisons, providing a quantitative and convenient tool for the optimal design, condition monitoring, and life prediction of rubber seals in hydropower units. The paper is organized as follows: the theoretical basis for characteristic physical quantities of aged rubber is first presented; a feature-extraction model for radial compression is then developed; finally, the practicality and accuracy of the method are verified by comparing measured data with empirical hardness formulas.

The significance of this study lies in transforming the complex material-aging assessment into a simple geometric measurement, providing a quantitative and practical tool for industrial applications. By integrating theoretical analysis from energy principles, numerical simulation of radial compression, and experimental validation using 17-year serviced specimens, this work establishes a robust framework that bridges microscopic energy storage degradation with macroscopic deformation parameters (*b*/*D*). Unlike conventional methods relying on hardness testing or destructive sampling, the proposed approach enables rapid, on-site evaluation of rubber seal conditions through a single geometric parameter. This methodology not only resolves the unclear aging mechanism under multi-factor coupling in hydropower units but also offers a solid theoretical foundation and practical solution for condition monitoring and life prediction of critical sealing components, demonstrating substantial engineering value for ensuring the reliability and economic efficiency of hydroelectric power station operations.

## 2. Theoretical Analysis of Aging-Characteristic Physical Quantities Based on Energy Principles

This section starts from the principle of energy conservation to theoretically derive the key physical quantities that characterize rubber aging. By analyzing the strain energy density function, it clarifies how the elastic modulus (*E*) and yield stress (*σ_s_*) represent the material’s energy storage capacity and the threshold for irreversible deformation, and it identifies the long-term aging trend of decreasing Mises stress alongside increasing elastic modulus. The core aim is to establish a theoretical link between aging and these physical quantities, thereby providing a foundation for applying the short-axis diameter ratio.

During long-term aging, rubber components that are subjected to loads and remain stationary undergo irreversible deformation, commonly referred to as “permanent deformation.” At the microscopic level, the cross-linked network within the rubber material changes, resulting in a weakening of its energy storage capacity. Therefore, the elastic deformation energy of stationary rubber material particles is analyzed. The strain energy density is expressed as

(1) $$V_{\varepsilon }=\frac{1}{2}\sigma_{1}\varepsilon_{1}+\frac{1}{2}\sigma_{2}\varepsilon_{2}+\frac{1}{2}\sigma_{3}\varepsilon_{3}$$

Among them, *σ_i_* (*i* = 1, 2, 3) are the three principal stresses and *ε_i_* (*i* = 1, 2, 3) are the corresponding three principal strains. Combined with the constitutive relationship, the above equation can also be expressed as

(2) $$V_{\varepsilon }=V_{v}+V_{d}$$

Among them, the body strain energy density is

(3) $$V_{v}=\frac{1-2\mu }{6E}{\left(\sigma_{1}+\sigma_{2}+\sigma_{3}\right)}^{2}$$

The distortion energy density is

(4) $$V_{d}=\frac{1+\mu }{6E}\left[{\left(\sigma_{1}-\sigma_{2}\right)}^{2}+{\left(\sigma_{2}-\sigma_{3}\right)}^{2}+{\left(\sigma_{3}-\sigma_{1}\right)}^{2}\right]=\frac{\sigma_{Mises}^{2}}{6G}$$

Among them, *E* is the elastic modulus, *G* is the shear modulus, *μ* is the Poisson’s ratio, and *σ_Mises_* is the Mises stress. Note that rubber is an incompressible material with a Poisson’s ratio of 0.5. Therefore, the strain energy density is

(5) $$V_{\varepsilon }=\frac{\sigma_{Mises}^{2}}{2E}$$

The above analysis indicates that the physical quantities used to measure the strain energy (or “energy storage capacity”) of rubber at different times (particularly during the long-term aging process) are solely the Mises stress and the elastic modulus. Qualitatively, during the long-term aging process, the elastic modulus increases, while the corresponding Mises stress decreases, resulting in a reduction in the total strain energy density and a weakening of the rubber’s energy storage capacity. It is important to note that as aging occurs, the Mises stress gradually decreases, which implies the existence of a certain limit (denoted as *σ_s_*, in this paper, referred to as the “yield stress”). When the Mises stress exceeds this limit (referred to as “yield stress,” *σ_s_*), the deformation becomes non-elastic. In this paper, non-elastic deformation is defined as “irreversible deformation” or “permanent deformation.” This means that after removing the external force, the material does not return to its original state. Consequently, no elastic energy is stored, and the energy is completely dissipated,

(6) $$d\varepsilon \left\{\begin{array}{l} >0,\sigma_{Mises}<\sigma_{s}\\ =0,\sigma_{Mises}=\sigma_{s} \end{array}\right.$$

Among them, d*ε* is the elastic strain increment. Thus, the two important physical quantities for measuring the long-term aging of rubber are its elastic modulus *E* and its yield stress *σ_s_*.

## 3. Establishment of a Radial Compression Feature-Extraction Model Based on the Short-Axis Diameter Ratio

In this chapter, a complete load–unload–reload simulation framework is built. This framework reproduces the aging process of rubber seals under service conditions. The first loading step represents long-term operation. During this phase, the strip sustains radial compression for years. The unloading step corresponds to the shutdown or removal stage. During this stage, permanent set develops. The subsequent reload step mimics laboratory testing. This testing is used to probe the aged material response.

Leveraging the energy-based physical quantities derived earlier, an aging-feature-extraction model centered on the short-axis diameter ratio *b*/*D* is established. Numerical simulations of the strip under 25% and 30% nominal compression trace the full mechanical history—initial loading, unloading, and secondary loading—to reveal how *b*/*D* evolves and how it correlates with mechanical parameters.

Quantitative relationships are then derived that link *b*/*D* to (i) the dimensionless yield stress *σ_s_*/*E* and (ii) the dimensionless unit-length reaction force *F*/*ED*. These closed-form connections provide a theoretical basis for rapid aging assessment from a simple geometric measurement.

To confirm model reliability, a dedicated experimental campaign is conducted: strips extracted from actual hydropower service are tested under the same reload protocol, and the data are systematically compared with simulation outputs. The close agreement validates the *b*/*D*-based feature-extraction approach and completes the roadmap from theory to field application, offering solid support for deploying the method in engineering practice.

### 3.1. Quantitative Relationship Between the Short-Axis Diameter Ratio and the Dimensionless Yield Stress

**Figure 1:**
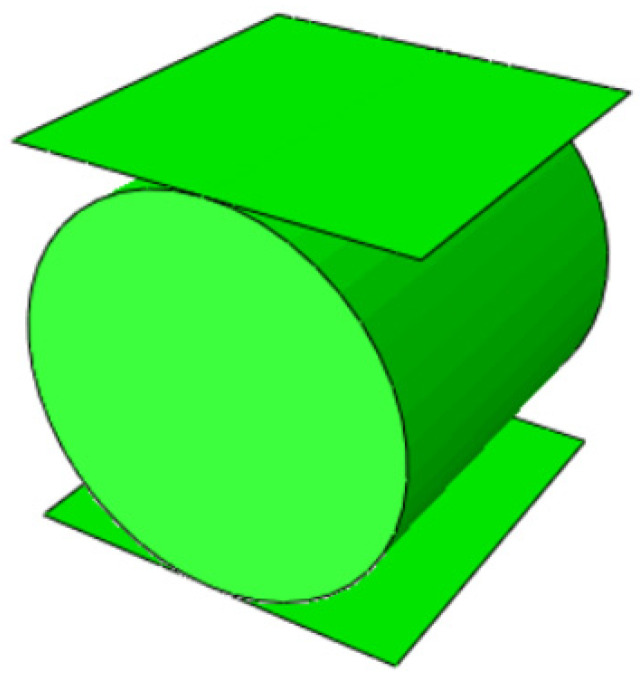
Schematic diagram of the model.

The following analysis examines the radial displacement load on rubber strips (a common operating condition for rubber products used as sealing components). The nominal compression ratio (the ratio of radial compression displacement to the original radial length) is 25% and 30%. The model diagram is shown in Figure 1.

**Figure 2:**
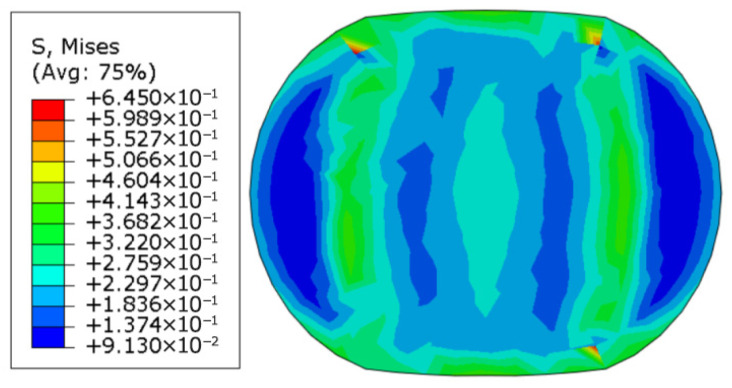
Cross-section stress diagram of a rubber strip model with yield stresses.

Figure 2 shows the residual Mises stress diagram for a cross-section with an original diameter of 9.5 mm, a Young’s modulus of 10 MPa, a yield stress of 1.0 MPa, and a residual stress after 25% compression and release of load. The model exhibits some irreversible deformation.

**Figure 3:**
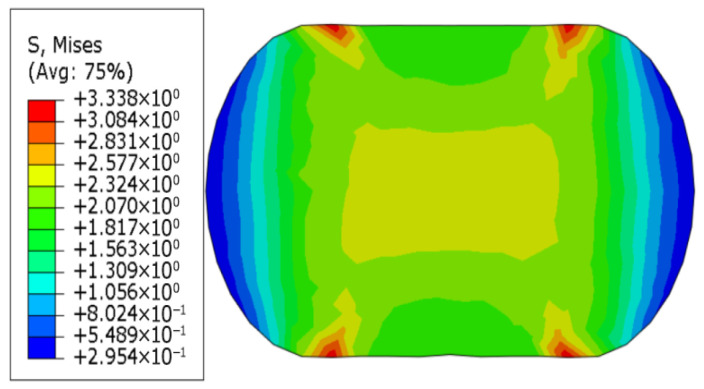
Consider continuing to load a 25% displacement model section after aging.

When there is no yield, the unit-length specimen reaction force at 25% compression is 11.6742 N/mm (when the modulus is 10.00 MPa), and the unit-length specimen reaction force at 30% compression is 15.3127 N/mm (when the modulus is 10.00 MPa). The original diameter is denoted as *D*, the horizontal axis passing through the center after deformation is the major axis a, and the vertical axis passing through the center after deformation is the minor axis *b*. In the figure above, the current configuration is subjected to a 25% load, i.e., after the (stiffer) structure contacts the rubber model, it continues to deform downward by 0.25*b* and maintains the deformation. The Mises stress distribution is shown in Figure 3.

Table 1 shows the major axis (the length of the longest line segment passing through the original center of the circle between two points on the edge of the cross-section when irreversible deformation occurs) and minor axis (the length of the shortest line segment passing through the original center of the circle between two points on the edge of the cross-section when irreversible deformation occurs) corresponding to different yield stresses when the compression ratio is 25%.

Similarly, Table 2 below shows the long axis and short axis corresponding to different yield stresses when the compression ratio is 30%.

Fit the long axis and short axis using the following formula:

(7) $${\left(\frac{a}{D}\right)}^{1-k}{\left(\frac{b}{D}\right)}^{k}=1$$

For a compression ratio of 25%, *k* = 0.2698 (R^2^ = 99.41%); for a compression ratio of 30%, *k* = 0.2987 (R^2^ = 97.6%).

Qualitatively speaking, the greater the yield stress, the longer the short axis. The following is a quantitative analysis of the relationship between yield stress and the short axis. If it is a uniaxial relationship, when the compression rate is 25% there are two cases: one is when the yield stress is 3.00 MPa, *σ_s_*/*E* = 0.30, *b*/*D* = 1.00; the other is when the yield stress is 0.00 MPa, and the 25% compression should be completely yielded, *b*/*D* = 0.75 (after removing the load, due to deformation coordination, this equation is not strictly equal). Therefore, the approximate formula for the yield stress and short axis under uniaxial conditions is as follows:

(8) $$\frac{\sigma_{s}}{E}=1.2\frac{b}{D}-0.9$$

The situation mentioned in this article is not a single-axis relationship. Referring to the form of the above equation and using the data in the table, for a compression ratio of 25%, linear fitting is performed, and the relationship between yield stress and short-axis length is as follows:

(9) $$\frac{\sigma_{s}}{E}=1.056\frac{b}{D}-0.7911$$

The goodness-of-fit R^2^ = 99.15%. Similarly, using the data in the table, for a compression ratio of 30%, linear fitting was performed, and the relationship between yield stress and short-axis length was analyzed.

(10) $$\frac{\sigma_{s}}{E}=1.035\frac{b}{D}-0.7092$$

The goodness-of-fit R^2^ = 99.82%.

### 3.2. Quantitative Relationship Between the Short-Axis Diameter Ratio and the Dimensionless Unit-Length Reaction Force

**Figure 4:**
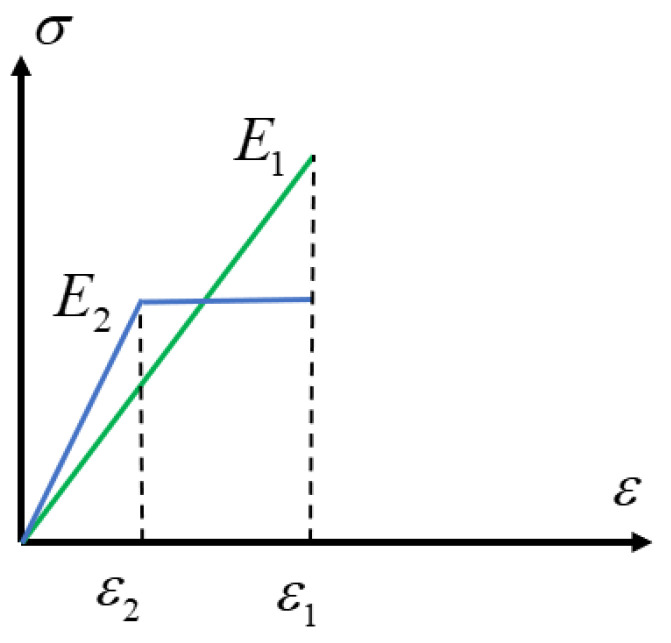
Schematic diagram of the resilient state and the resilient–inelastic state.

The following analysis considers the case of aged rubber subjected to additional loading, examining both “purely elastic” and “elastic-inelastic” states [19], as shown in Figure 4.

State 1 is a purely elastic state, and the strain energy is

(11) $$V_{1}=\frac{1}{2}E_{1}\varepsilon_{1}^{2}$$

State 2 is the ideal elastic–plastic state, and the strain energy is

(12) $$V_{2}=E_{2}\varepsilon_{2}\varepsilon_{1}-\frac{1}{2}E_{2}\varepsilon_{2}^{2}$$

And there is energy loss, so there should be

(13) $$V_{2}=\eta V_{1}$$

where *η* is the ratio of the elastic properties after aging to the initial elastic properties, which is generally less than 1.00. Furthermore,

(14) $$\frac{\varepsilon_{2}}{\varepsilon_{1}}=1-\sqrt{1-\frac{\eta E_{1}}{E_{2}}}$$

There is an (approximate) proportional relationship between the elasticity of the initial state and the current state:

(15) $$E_{1}∼\frac{F}{D}$$

Or it can be expressed as

(16) $$E_{1}=\gamma \frac{F}{D}$$

Among them, *γ* is the coefficient. The modulus of the current state is

(17) $$E_{2}=E$$

where *E* is the elastic modulus after aging, which should generally be greater than the initial elastic modulus *E*_0_.

In addition, there is the following direct proportional relationship:

(18) $$\frac{\varepsilon_{2}}{\varepsilon_{1}}∼\frac{b}{D}$$

Or it can be expressed as

(19) $$\frac{\varepsilon_{2}}{\varepsilon_{1}}=\kappa \frac{b}{D}$$

Among them, *κ* is the coefficient. Therefore, we have

(20) $$\eta \gamma \frac{F}{ED}=-\kappa^{2}{\left(\frac{b}{D}\right)}^{2}+2\kappa \left(\frac{b}{D}\right)$$

That is

(21) $$\frac{F}{ED}=-\frac{\kappa^{2}}{\eta \gamma }{\left(\frac{b}{D}\right)}^{2}+\frac{2\kappa }{\eta \gamma }\left(\frac{b}{D}\right)$$

Based on the above formula, for a model with an initial compression rate of 25% that has undergone aging, continue loading by 25%. The elastic modulus during the first loading is adjusted to 0.00001 MPa. The elastic modulus during the second loading is adjusted to 10.00 MPa. This ensures that the ratio of yield stress to elastic modulus remains constant between the two loadings. Such adjustments result in residual stress from the first loading being extremely small. Thus, this residual stress is negligible. Only residual strain is retained. In other words, only “permanent deformation” is retained. Residual stress is not retained. Consequently, the final force from the second loading is significantly influenced by the current state. It is not influenced by residual stress from the first loading. The loading displacement and contact force per unit length after loading are shown in Table 3.

Use a formula similar to Equation (21) for fitting:

(22) $$\frac{F}{ED}=-0.4209{\left(\frac{b}{D}\right)}^{2}+0.5494\left(\frac{b}{D}\right)$$

The goodness of fit is R^2^ = 98.66%. Similarly, for the model that aged at an initial compression rate of 30%, continue to load 30% (the elastic modulus of the two loads is adjusted in the same way as when the compression rate is 25%). The loading displacement and contact force per unit length after loading are shown in Table 4.

Use a formula similar to Equation (21) for fitting:

(23) $$\frac{F}{ED}=-0.4439{\left(\frac{b}{D}\right)}^{2}+0.6174\left(\frac{b}{D}\right)$$

The goodness of fit is R^2^ = 99.73%.

Combining the above two equations, the parameters corresponding to Equation (21). are shown in Table 5.

### 3.3. Experimental Validation and Simulation Comparison of the Reload Model

**Figure 5:**
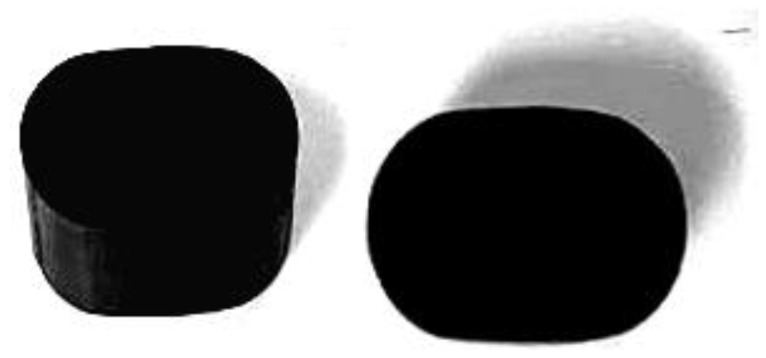
Truncated specimens of rubber strips (*No*. 0) that have been in service for a long time under load.

Based on the above model, the following analysis is conducted on unaged and aged rubber strips (cut samples). The cross-sectional diameter of the unaged rubber strip is 9.50 mm, while the long axis a of the aged rubber strip is 13.52 mm and the short axis b is 10.82 mm. The three-dimensional view and top view of the aged rubber strip sample are shown in Figure 5.

**Figure 6:**
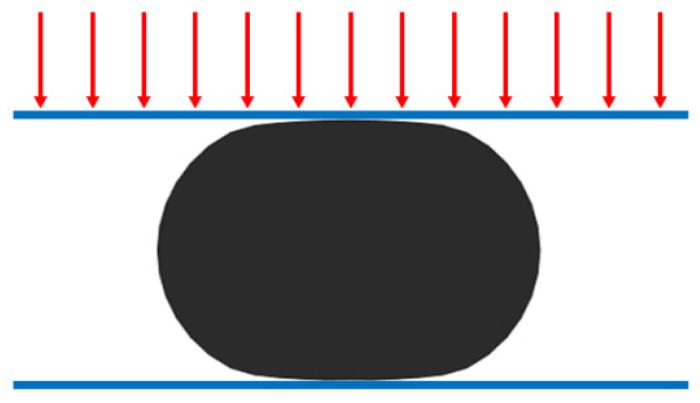
Schematic diagram of the applied load applied to a rubber strip truncated specimen that has been in service for a long time under load.

Combining Equation (7), the original cross-sectional diameter of the aged rubber strip is obtained as *D* = 12.65 mm (30% nominal compression ratio). *b*/*D* = 0.8553. Combining Equation (10), *σ_s_*/*E* = 0.1760 is obtained. For the unaged and aged samples, a universal tensile and compression testing machine is used to apply a 30% vertical load, as shown in Figure 6 (the red arrow represents the externally applied displacement load or force load).

For unaged material specimens, the force per unit length of the specimen when compressed by 30% is 11.45 N/mm. Note that when there is no yield, the reaction force per unit length of the specimen compressed by 30% is 15.3127 N/mm (when the modulus is 10 MPa), so the elastic modulus *E*_0_ of the unaged specimen is *E*_0_ = 11.45/15.3127 × 10 MPa = 7.5 MPa. When conducting uniaxial compression tests on corresponding material samples, the modulus obtained was 7.8 MPa, with a difference of only 3.85% between the two values.

For aged material specimens, the force per unit length of the specimen when compressed by 30% is *F* = 40.34 N/mm. Taking *b* = 10.82 mm and *D* = 12.65 mm, and combining with Equation (23), *E* = 15.7 MPa. Combining with *σ_s_*/*E* = 0.1760, the yield stress is obtained as *σ_s_* = 2.76 MPa.

**Figure 7:**
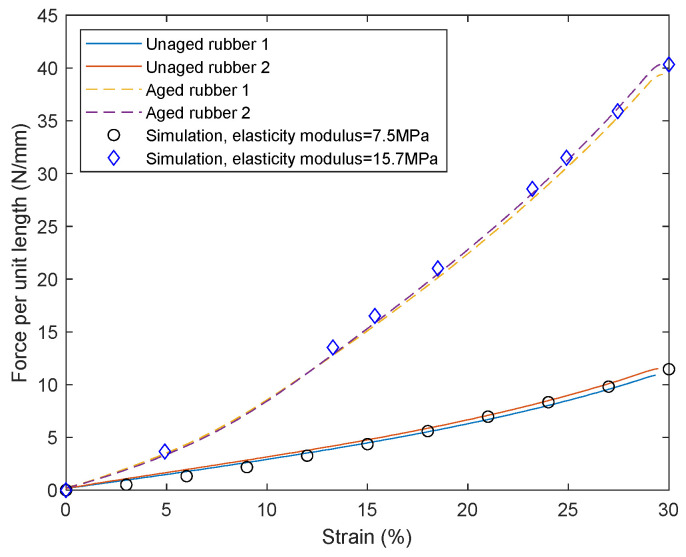
Comparison of test and simulation results.

**Figure 8:**
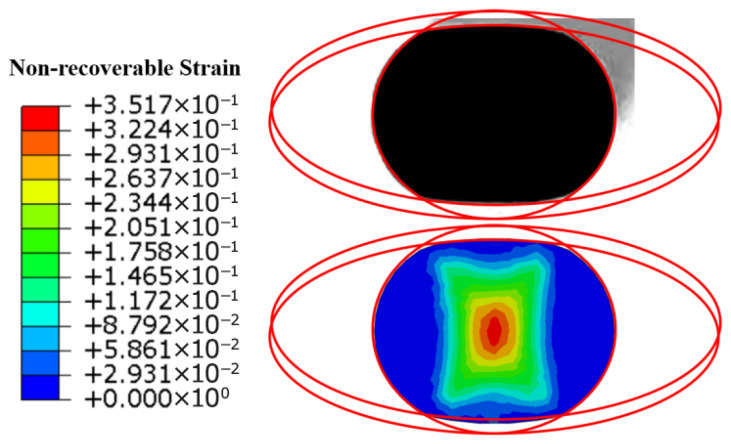
Actual and simulated cross-sections of aging specimens.

For unaged FKM specimens, simulation analysis was performed using *E* = 7.5 MPa; for aged NBR specimens, simulation analysis was performed using *E* = 15.7 MPa and *σ_s_* = 2.76 MPa. The comparison of experimental and simulated results is shown in Figure 7:

The cross-sections of the aged specimens and the cross-sections after simulated aging unloading are shown in Figure 8 (the red line represents a part of the ellipse and depicts the outer edge of the cross-sectional shape of the sample with aged rubber strip truncation).

The actual cross-section of the aged sample shown in the figure above is consistent with the simulated cross-section. The simulation shows that the maximum irreversible deformation occurs at the center of the rubber strip cross-section, which is 0.3517.

## 4. Experimental Validation and Comparative Analysis of Aging Features in Service-Exposed Rubber Strips

In this chapter, the aging-feature-extraction model is evaluated against real engineering samples. This model is based on the short-axis diameter ratio. It was developed in Chapter 2. Strips of fluororubber (FKM) and nitrile–butadiene rubber (NBR) were collected. These strips served for 17 years. Systematic experiments were conducted. Data analyses were also performed. These steps verify the validity of *b*/*D* as an aging indicator. A universal tension–compression machine was employed to perform radial compression tests and obtain mechanical parameters. The elastic moduli back-calculated from *b*/*D* were then compared with estimates derived from hardness-based empirical correlations (Gent and Qi formulas).

**Figure 9:**
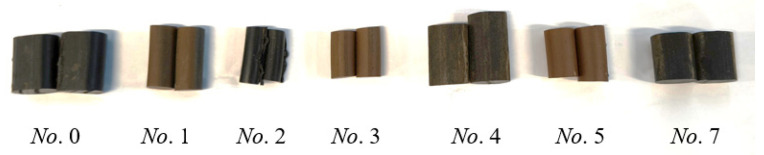
Aged rubber strip specimens.

Based on the above process, samples of several rubber strips that had been in use for a long time were analyzed, as shown in Figure 9.

Sample *A* refers to the unaged sample in the aforementioned analysis process, while sample 0 refers to the aged sample in the aforementioned analysis process. The basic information of other aged samples is shown in Table 6 below (text or values underlined indicate missing original data, which has been supplemented or estimated based on various factors):

The geometric information and service life of each rubber material are shown in Table 7. The values for *D*_0_, *a*, and *b* were obtained by taking the average of multiple measurements using a Vernier caliper. *D* is the diameter calculated using Equation (7) (some data is slightly larger than the design diameter, due to factors such as long-term swelling of the rubber), the compression ratio is calculated using *b* and *D* (distinct from the nominal compression ratio; if this value cannot be calculated, the nominal compression ratio can be used as an alternative), and the service life is the actual number of years the rubber has been in use, rounded to the nearest whole number.

**Figure 10:**
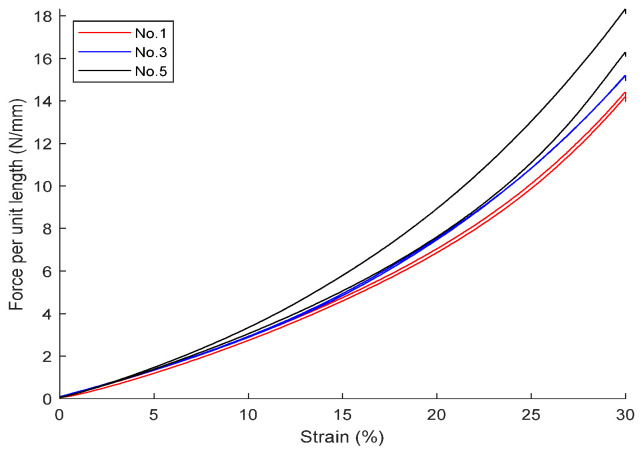
Radial compression results of aged fluororubber (FKM).

**Figure 11:**
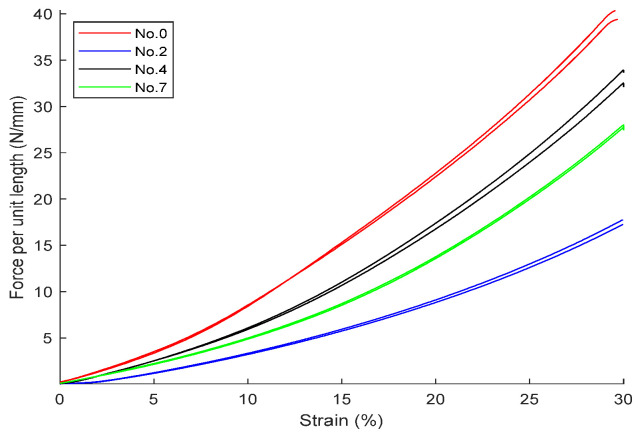
Results of radial compression of aged nitrile rubber (NBR).

Using a universal tensile and compression testing machine, compression tests were conducted on the above-mentioned aged samples. The results for fluororubber and nitrile rubber are shown in Figure 10 and Figure 11.

Combining the *b*/*D* and compression ratio, use Equations (9) or (10) to calculate the *σ_s_*/*E*. Note that the compression ratios of nitrile rubber materials (numbers 0, 2, 4, and 7) are mostly between 25% and 30%. Combining Equation (22) (compression ratio 25%) and Equation (23) (compression ratio 30%), use linear interpolation to give the following estimated formula for a compression ratio of 27.5%:

(24) $$\frac{F}{ED}=-0.4324{\left(\frac{b}{D}\right)}^{2}+0.5834\left(\frac{b}{D}\right)$$

Using the above equation, combined with the pressure per unit length from the two figures mentioned above, the modulus *E* can be calculated (in actual engineering practice, for convenience of calculation, Equations (22) or (23) can also be directly used), and subsequently the yield stress *σ_s_* can be calculated. Using a hardness tester, measure its Shore A hardness, and combine it with the empirical formulas from Gent [20] and Qi [21] to calculate the corresponding estimated value of the elastic modulus; generally, when calculating the elastic modulus of rubber using measured hardness, the value should fall between the two empirical formulas, and for specific materials, there should be a specific trend curve.

**Figure 12:**
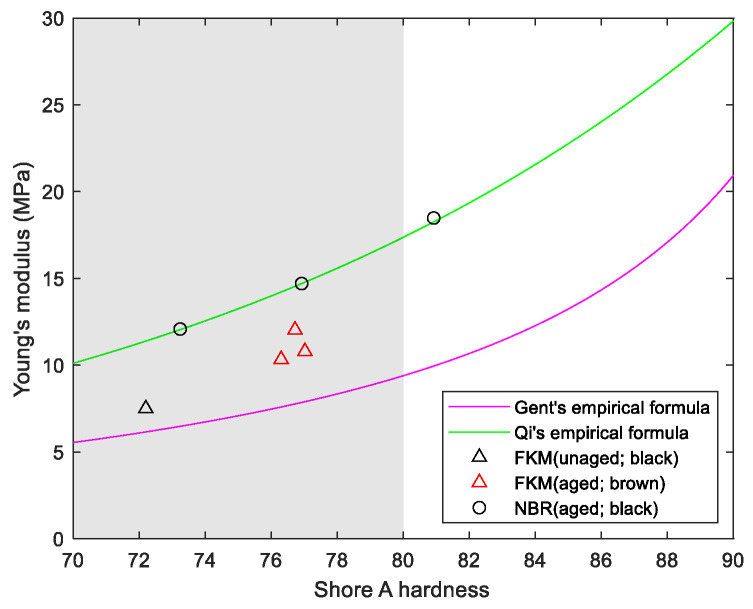
Comparison of the modulus of an aged material with an estimate of the modulus calculated using hardness.

The yield stress and modulus of various rubber materials are shown in Table 8 (where yield stress is infinite, indicating the material is in a purely elastic state with no permanent deformation). The comparison chart between the modulus of aged materials and the estimated modulus calculated based on service hardness is shown in Figure 12 (Shore A hardness of 70–80 is the specified requirement for new rubber strips under specific operating conditions). As shown in the figure, the modulus calculated using the hardness of unaged fluorocarbon rubber or aged fluorocarbon rubber (but without permanent deformation) falls between the empirical formulas of Gent and Qi. The modulus calculated using the hardness of aged nitrile rubber (with significant permanent deformation) aligns well with Qi’s empirical formula.

## 5. Conclusions

This paper systematically proposes and validates an aging-feature-extraction methodology. This methodology uses the short-axis diameter ratio (*b*/*D*) of service-exposed rubber strips. The work integrates theoretical analysis, numerical simulation, and experimental verification. Consequently, an aging assessment framework is established. This framework is centered on a single geometric parameter. Starting from energy principles, elastic modulus (*E*) and yield stress (*σ_s_*) are identified as the key physical descriptors of rubber aging. Radial compression simulations then reveal a linear correlation between *b*/*D* and the dimensionless yield stress (*σ_s_*/*E*) and a quadratic correlation between *b*/*D* and the dimensionless unit-length reaction force (*F*/*ED*), laying a solid theoretical foundation for quantitative aging characterization.

Reload tests demonstrate that the mechanical response of aged rubber under subsequent loading is captured by the model with high fidelity. For compression levels of 25% and 30%, the coefficient of determination between experimental and predicted force–displacement curves exceeds 98%, confirming the reliability of the approach.

Comparative tests on field samples—both FKM and NBR seals after 17 years of service—show that elastic moduli back-calculated from *b*/*D* agree well with estimates from hardness-based empirical formulas (Gent and Qi). NBR results in particular align closely with the Qi formula, further underscoring the method’s practicality and accuracy.

By converting the complex problem of material aging into the measurement of a readily accessible geometric parameter, the approach enables rapid, quantitative, and user-friendly condition assessment of rubber seals. The findings provide an effective tool for condition monitoring, life prediction, and maintenance management of industrial sealing components, especially for hydropower units operating in demanding environments, and offer significant theoretical and practical value for broader engineering applications.

## Figures and Tables

**Table 1:** The compression rate is 25% for short-axis data.

*σ_s_*/MPa	*D*/mm	*a*/mm	*b*/mm
1.00	9.50	10.11	7.99
1.40	9.50	9.96	8.38
1.80	9.50	9.81	8.75
2.20	9.50	9.66	9.10
2.60	9.50	9.53	9.36
3.00	9.50	9.52	9.47

**Table 2:** The compression rate is 30% for short-axis data.

*σ_s_*/MPa	*D*/mm	*a*/mm	*b*/mm
1.00	9.50	10.46	7.45
1.40	9.50	10.33	7.80
1.80	9.50	10.17	8.18
2.20	9.50	9.99	8.55
2.60	9.50	9.82	8.89
3.00	9.50	9.66	9.18

**Table 3:** The contact force of 25% of the displacement load is applied after 25% of the displacement load is removed.

*σ*_*s*_/*E*	25% Displacement After Aging (mm)	Contact Force *F* After Loading (N/mm)
0.10	2.00	15.57
0.14	2.09	14.87
0.18	2.19	14.24
0.22	2.27	13.49
0.26	2.34	12.43
0.30	2.37	11.73

**Table 4:** The contact force of 30% of the displacement load is applied after 30% of the displacement load is removed.

*σ*_*s*_/*E*	30% Displacement After Aging (mm)	Contact Force *F* After Loading (N/mm)
0.10	2.24	20.07
0.14	2.34	19.75
0.18	2.45	19.28
0.22	2.57	18.60
0.26	2.67	17.97
0.30	2.75	17.15

**Table 5:** Reload the formula parameter table.

Parameters	When the Compression Ratio Is 25%	When the Compression Ratio Is 30%
κ	1.532	1.438
ηγ	5.577	4.658

**Table 6:** Rubber material information sheet.

Number	Density (g/cm^3^)	Rubber Type	Exterior Color	Groove Type	Both Sides of the Medium	Design Diameter (mm)	Groove Height (mm)	Nominal Compression Ratio
*A*	1.86	FKM	Black	/	/	/	/	/
0	/	NBR	Black	Rectangular groove	Water–air	12	9	25.00%
1	2.04	FKM	Brown	Triangular groove	Oil–water	8	6	25.00%
2	1.29	NBR	Black	Rectangular groove	Oil–oil	6	4.5	25.00%
3	2.04	FKM	Brown	Rectangular groove	Oil–oil	8	6	25.00%
4	1.31	NBR	Black	Rectangular groove	Water–air	12	9	25.00%
5	2.01	FKM	Brown	trapezoidal groove	Oil–air	10	7	32.56%
7	1.30	NBR	Black	Rectangular groove	Water–air	12	9	25.00%

**Table 7:** Summary table of geometric information and service life of each rubber.

Number	Diameter *D*_0_ or Major Axis Length *a* (mm)	Short Shaft Length *b* (mm)	Calculated Diameter *D* (mm)	Compression Ratio	Service Life (Years)	*b*/*D*
*A*	9.5000	/	/	30%	/	/
0	13.520	10.820	12.650	28.85%	17	0.855
1	8.214	/	/	/	17	/
2	5.984	5.672	5.898	23.71%	17	0.962
3	7.828	/	/	/	17	/
4	12.960	10.312	12.185	26.14%	17	0.846
5	10.380	/	/	/	17	/
7	13.228	10.536	12.440	27.65%	17	0.847

**Table 8:** Summary table of material information and service life of each rubber.

Number	*b*/*D*	*σ_s_*/*E*	Modulus *E* (MPa)	Yield Stress (MPa)	Hardness (Shore A)	Estimated Elastic Modulus (Gent)	Estimated Elastic Modulus (Qi)
*A*	/	/	7.5	inf	72.2	6.15	11.39
0	0.855	0.176	15.684	2.760	/	/	/
1	/	/	10.794	inf	77.02	7.90	14.78
2	0.962	0.286	18.477	5.286	80.92	9.95	18.25
3	/	/	12.031	inf	76.72	7.77	14.54
4	0.846	0.103	14.699	1.508	76.92	7.85	14.70
5	/	/	10.336	inf	76.3	7.59	14.22
7	0.847	0.103	12.080	1.247	73.24	6.48	12.05

## Data Availability

The data underlying this study contain sensitive information. Therefore, the data cannot be made publicly available.

## References

[1] de Santis R.B., Gontijo T.S., Costa M.A. (2022). Condition-based maintenance in hydroelectric plants: A systematic literature review. Proc. Inst. Mech. Eng. Part O J. Risk Reliab..

[2] Xu L., Zeng B., Wang C., Zhang F. (2020). Current Status and Prospects for the Development of High-Performance Synthetic Rubber Materials in China. Chin. J. Eng. Sci..

[3] Chen B., Dai J., Song T., Guan Q. (2022). Research and development of high-performance high-damping rubber materials for high-damping rubber isolation bearings: A review. Polymers.

[4] Wei Z., Chen H. (2019). A new elastic constitutive model for rubber materials. J. Mech..

[5] Park J., Lee B., Kim H.J. (2022). A comparative study on the physical properties of a fluoro rubber complex. Mater. Sci. Technol..

[6] Grasland F., Chazeau L., Chenal J.M. (2019). About thermo-oxidative ageing at moderate temperature of conventionally vulcanized natural rubber. Polym. Degrad. Stab..

[7] Guo Q., Shao H. (2022). Research progress on the aging mechanism and aging behavior of rubber. Polym. Bull..

[8] Zhang H., Han W., Cheng Z., Fan W., Long H., Liu Z., Zhang G. (2022). Study on the thermal-oxidative aging mechanism of modified steel slag/rubber composite materials based on SEM and FTIR. Spectrosc. Spectr. Anal..

[9] Li Z.X., Kong Y.R., Chen X.F., Huang Y.J., Lv Y.D., Li G.X. (2023). High-temperature thermo-oxidative aging of vulcanized natural rubber nanocomposites: Evolution of microstructure and mechanical properties. Chin. J. Polym. Sci..

[10] Klüppel M., Jungk J. (2022). Thermo-oxidative aging and mechanical fatigue of elastomer compounds used in various fields of rubber industry. Degradation of Elastomers in Practice, Experiments and Modeling.

[11] Simon A., Pepin J., Berthier D., Méo S. (2023). Degradation mechanism of FKM during thermo-oxidative aging from mechanical and network structure correlations. Polym. Degrad. Stab..

[12] Liu Y., Yu J., Zhang Q. (2026). Aging and life assessment of rubber. Mater. Rev..

[13] Hong D., Ma Y., Zhao G. (2022). Experimental study on the mechanical properties of natural rubber under alternating freeze-thaw cycles and thermal aging. Vib. Shock..

[14] Kong P., Chen X., Zhuo S., Leng Z., Xu G., Shen K., Wang S., Zhou Y., Teng G., Yang J. (2024). Thermo-oxidative aging performance of mechanochemical activated rubber powder modified asphalt. Constr. Build. Mater..

[15] Lou W., Zhang W., Jin T., Liu X., Wang H. (2019). Stress–thermal oxidative aging behavior of hydrogenated nitrile rubber seals. J. Appl. Polym. Sci..

[16] Dong L., Li K., Zhu X., Li Z., Zhang D., Pan Y., Chen X. (2020). Study on high temperature sealing behavior of packer rubber tube based on thermal aging experiments. Eng. Fail. Anal..

[17] Li Y., Wu J., Chen Z., Zhang Z., Su B., Wang Y. (2024). The Influence of Oil and Thermal Aging on the Sealing Characteristics of NBR Seals. Polymers.

[18] Kömmling A., Jaunich M., Wolff D. (2016). Effects of heterogeneous aging in compressed HNBR and EPDM O-ring seals. Polym. Degrad. Stab..

[19] Meng C.Y. (2024). An anisotropic finite strain elastoplastic model considering different plastic spin effects on the intermediate configuration. Mech. Res. Commun..

[20] Gent A.N. (1958). On the Relation between Indentation Hardness and Young’s Modulus. Rubber Chem. Technol..

[21] Qi H.J., Joyce K., Boyce M.C. (2003). Durometer Hardness and the Stress-Strain Behavior of Elastomeric Materials. Rubber Chem. Technol..

